# Extracellular vesicles miR-574-5p and miR-181a-5p as prognostic markers in NSCLC patients treated with nivolumab

**DOI:** 10.1007/s10238-024-01427-8

**Published:** 2024-08-06

**Authors:** Carlo Genova, Silvia Marconi, Giovanna Chiorino, Francesca Guana, Paola Ostano, Sara Santamaria, Giovanni Rossi, Irene Vanni, Luca Longo, Marco Tagliamento, Lodovica Zullo, Maria Giovanna Dal Bello, Chiara Dellepiane, Angela Alama, Erika Rijavec, Vienna Ludovini, Giulia Barletta, Francesco Passiglia, Giulio Metro, Sara Baglivo, Rita Chiari, Licia Rivoltini, Federica Biello, Iosune Baraibar, Ignacio Gil-Bazo, Silvia Novello, Francesco Grossi, Simona Coco

**Affiliations:** 1 UOC Clinica Di Oncologia Medica, IRCCS Ospedale Policlinico San Martino, Largo Rosanna Benzi, 10, 16132 Genoa, Italy; 2 Dipartimento Di Medicina Interna E Specialità Mediche (DiMI), Università Degli Studi Di Genova, Viale Benedetto XV, 6, 16132 Genoa, Italy; 3 UOS Tumori Polmonari, IRCCS Ospedale Policlinico San Martino, Largo Rosanna Benzi, 10, 16132 Genoa, Italy; 4 Laboratory of Cancer Genomics, Fondazione Edo Ed Elvo Tempia, Via Malta, 3, 13900 Biella, Italy; 5 UOC Oncologia Medica 2, IRCCS Ospedale Policlinico San Martino, Largo Rosanna Benzi, 10, 16132 Genoa, Italy; 6 Genetica Oncologica, IRCCS Ospedale Policlinico San Martino, Largo Rosanna Benzi, 10, 16132 Genoa, Italy; 7 Medical Oncology Unit, Fondazione IRCCS Ca’ Granda Ospedale Maggiore Policlinico, Via Francesco Sforza, 35, 20122 Milan, Italy; 8 Department of Medical Oncology, Santa Maria Della Misericordia Hospital, Piazzale Giorgio Menghini, 3, 06129 Perugia, Italy; 9 Department of Oncology, University of Turin, S. Luigi Gonzaga Hospital, Regione Gonzole, 10, 10043 Orbassano, TO Italy; 10 Azienda Ospedaliera “Ospedali Riuniti Marche Nord”, Piazzale Cinelli 4, 61126 Pesaro, PU Italy; 11 Unit of Immunotherapy, Department of Research, Fondazione IRCCS Istituto Nazionale Dei Tumori, Via Giacomo Venezian, 1, 20133 Milan, Italy; 12 Oncology Unit, Azienda Ospedaliera Universitaria Maggiore Della Carità, Largo Bellini, 28100 Novara, Italy; 13 Department of Oncology, Clínica Universidad de Navarra, Av. de Pío XII, 36, 31008 Pamplona, Spain; 14 Program in Solid Tumors, Center for Applied Medical Research and Navarra Institute for Health Research, Av. de Pío XII, 55, 31008 Pamplona, Navarra Spain; 15 Centro de Investigación Biomédica en Red de Cáncer (CIBERONC), Av. Monforte de Lemos, 3-5, Pabellón 11, Planta 0, 28029 Madrid, Spain; 16 Division of Medical Oncology, Department of Medicine and Surgery, Ospedale Di Circolo E Fondazione Macchi, ASST Dei Sette Laghi, Via Lazio, 36, 21100 Varese, Italy

**Keywords:** Non-small cell lung cancer (NSCLC), Immune checkpoint inhibitor, Nivolumab, Extracellular vesicle miRNA, Prognosis score, miR-574-5p and miR-181a-5p, Pluripotency of stem cell, Toll-like receptor

## Abstract

Immune checkpoint inhibitors (ICIs) have revolutionized the management of advanced non-small cell lung cancer (NSCLC), although patient survival is still unsatisfactory. Accurate predictive markers capable of personalizing the treatment of patients with NSCLC are still lacking. Circulating extracellular vesicles involved in cell-to-cell communications through miRNAs (EV-miRs) transfer are promising markers. Plasma from 245 patients with advanced NSCLC who received nivolumab as second-line therapy was collected and analyzed. EV-miRnome was profiled on 174/245 patients by microarray platform, and selected EV-miRs were validated by qPCR. A prognostic model combining EV-miR and clinical variables was built using stepwise Cox regression analysis and tested on an independent patient cohort (71/245). EV-*PD-L1* gene copy number was assessed by digital PCR. For 54 patients with disease control, EV-miR changes at best response *versus* baseline were investigated by microarray and validated by qPCR. EV-miRNome profiling at baseline identified two EV-miRs (miR-181a-5p and miR-574-5p) that, combined with performance status, are capable of discriminating patients unlikely from those that are likely to benefit from immunotherapy (median overall survival of 4 months or higher than 9 months, respectively). EV-PD-L1 digital evaluation reported higher baseline copy number in patients at increased risk of mortality, without improving the prognostic score. Best response EV-miRNome profiling selected six deregulated EV-miRs (miR19a-3p, miR-20a-5p, miR-142-3p, miR-1260a, miR-1260b, and miR-5100) in responding patients. Their longitudinal monitoring highlighted a significant downmodulation already in the first treatment cycles, which lasted more than 6 months. Our results demonstrate that EV-miRs are promising prognostic markers for NSCLC patients treated with nivolumab.

**Supplementary Information:**

The online version contains supplementary material available at 10.1007/s10238-024-01427-8.

## Introduction

In recent years, immunotherapy has emerged as a valid treatment option in different types of cancer, including non-small cell lung cancer (NSCLC) [1, 2]. Nivolumab, an immune checkpoint inhibitor (ICI) directed against the programmed cell death-1 (PD-1) protein, is currently available as a further line of treatment for NSCLC patients with either squamous or non-squamous histology [1, 3]. However, despite the impressive results achieved by ICIs, a non-negligible fraction of patients does not benefit from immunotherapy; indeed, with specific regard to pre-treated patients, single-agent ICIs achieve objective response only in 20% of cases. The tumor expression of programmed cell death-1 ligand (PD-L1) has a predictive role in patients with NSCLC treated with ICIs, although its strength in second-line settings is less defined [1, 4, 5]. Therefore, the identification of more robust biomarkers for ICI is of the utmost importance. Circulating molecules are a promising source of non-invasive prognostic markers, especially in ICI-based regimes where immunocompetent cells participate in the response to the drug [6, 7]. In this regard, a number of blood miRNA-based signatures have been proposed as response predictors [8–10]. However, circulating extracellular vesicle microRNAs (EV-miRs) appear to be a better biomarker source than their cell-free counterparts, due to their stability, quantity, and quality [11]. Furthermore, evidence also indicates that tumor-derived EV-miRs can modulate the behavior of recipient cells [12]. In particular, EV-miRs have also been described to play a relevant role in the anti-tumor immune response, by immune cell modulation, as well as tumor antigen processing [13]. All previous evidence demonstrates that miRs, trapped in EVs, represent a surrogate for the tumor microenvironment and are promising prognostic markers, particularly in patients receiving ICIs, where both the immune system and the features of the tumor influence response to therapy. To date, few studies have reported on EV-miRs as predictors of response to ICIs in NSCLC [14–16], mainly including small cohorts of patients treated with both PD-1 or PD-L1-based therapies as a first or second line, with consequent limited clinical application. Recently, EV-PD-L1 (EV-PD-L1) mRNA copy number (CN) has also been described as an unfavorable prognostic marker in ICIs [17]. However, similar to the previous EV-miR studies, the NSCLC cohort was too limited to provide reliable results.

**Fig. 1:**
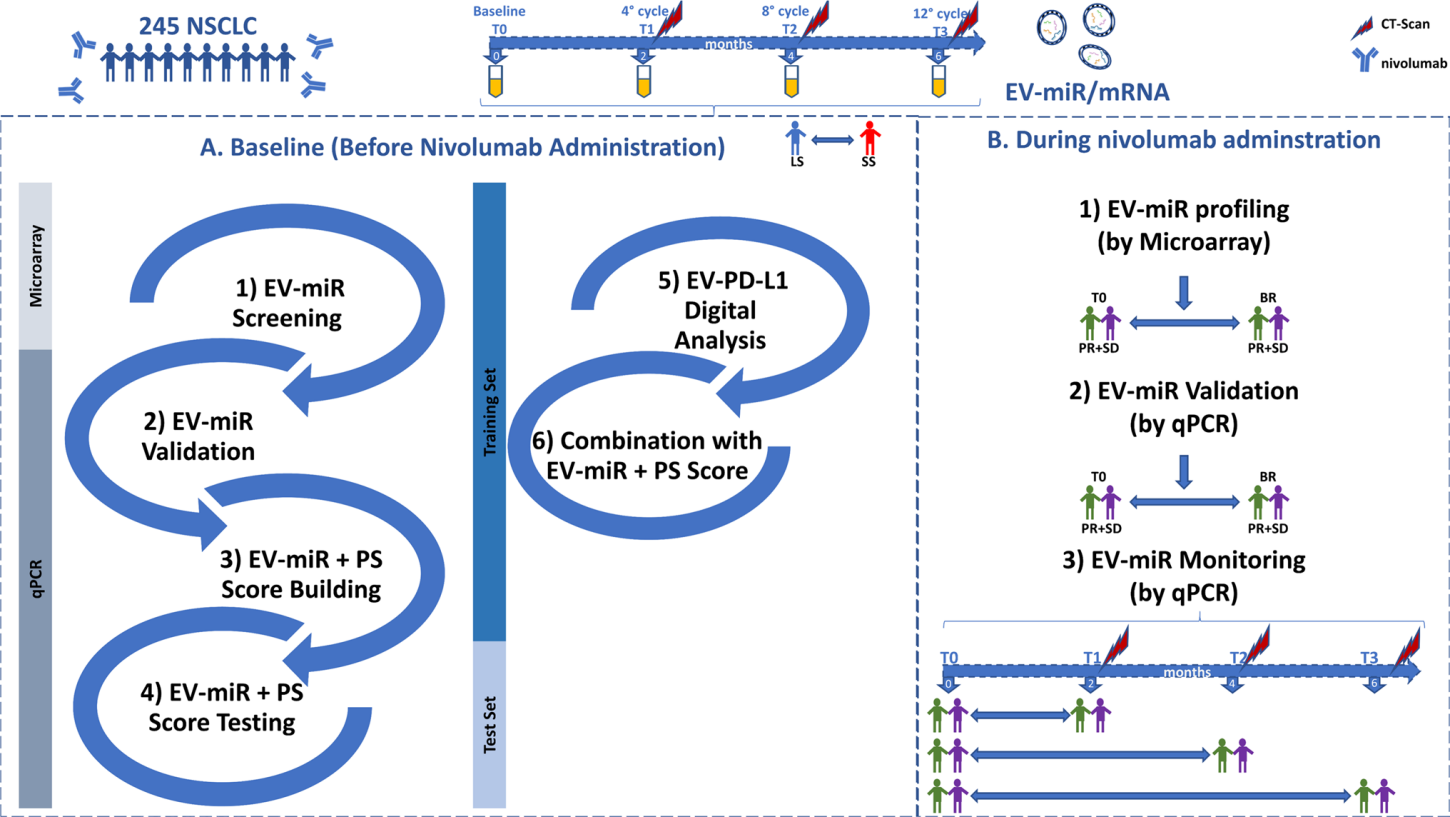
Graphical representation of the study design. *BR* best response, *LS* long survivors, *PR* partial response, *PS* performance status, *SD* stable disease, and *SS* short survivors

Here, we developed a robust EV-miR prognostic score in a large cohort of patients treated with nivolumab as second-line treatment for advanced NSCLC to select patients who would benefit most from treatment with this immunomodulator (Fig. 1a). We also evaluated the prognostic role of EV-PD-L1 CN mRNA assessed by digital PCR in the plasma of these patients. Finally, we demonstrated that some EV-miRs are deregulated in patients with disease control at the time of best response (BR). Notably, their modulation occurs at the first assessment, remaining deregulated 6 months after initiation of therapy, confirming a potential role in the immune cell modulation (Fig. 1B).

## Materials and methods

### Study population

From May 2015 to December 2022, a total of 245 patients with advanced NSCLC, who received nivolumab in second or subsequent lines of treatment, were enrolled from three cancer centers. Patients’ eligibility criteria were as follows: (i) histologically confirmed diagnosis of advanced NSCLC; (ii) disease progression after at least one line of platinum-based chemotherapy; (iii) no previous treatment with other ICIs; and (iv) no corticosteroid treatment at dose > 10 mg/day. The enrolled patients received nivolumab according to the indications based on the CheckMate 017 [1] and CheckMate 057 [3] studies and underwent computed tomography scan (CT scan) assessment every 4 cycles of therapy. Initially, nivolumab was administered at the dose of 3 mg/Kg every 2 weeks. However, starting from March 2, 2018, the dosing schedule was modified to a flat dose of 240 mg every 2 weeks, for the patients already on treatment as well as for new patients starting treatment thereafter. Nivolumab was administered until disease progression, onset of unacceptable toxicities, patient’s refusal, death, or up to 96 weeks of treatment. Treatment beyond tumor progression was allowed based on investigators’ judgment as long as clinical benefit was perceived. Disease progression (PD), stable disease (SD), and partial response (PR) were determined based on the Response Evaluation Criteria in Solid Tumors (RECIST) *v.1.1*. For each patient, a peripheral blood sample was collected at baseline and every 4 cycles of treatment, based on the patient’s status. In addition, 6 mL of peripheral blood samples in an EDTA tube were collected from 24 healthy individuals (18 males and six females). Plasma samples were isolated within 1 h from sampling by two consecutive rounds of centrifugation and stored at − 80 °C until the EV-miR isolation.

### EV characterization and EV-miR profiling

EV-RNA, including miRs, was isolated from frozen plasma stored at − 80 °C for an average of 15 months (0–37 months), without prior thawing (Supplementary Table S1). This cryopreservation approach ensured the preservation of both EV morphology and miR cargo integrity as previously demonstrated [18–20]. Subsequently, EV-miRs were purified using the Exo-RNeasy Midi Kit (Qiagen, Hilden, Germany) with the addition of a spike-in miR control (UniSP6, Qiagen), and their concentration was assessed by Qubit™ fluorometer using microRNA Assay Kit (Thermo Fisher Scientific, San Jose, CA, USA). To confirm the isolation of pure EVs, their size and concentration were investigated by nanoparticle tracking analysis (NTA) (NanoSight LM10, Malvern Instruments Ltd., Malvern, UK) and confirmed by both non-conventional cytofluorimetry (CFDA-SE Vybrant™ CFDA-SE Cell Tracer Kit, Thermo Fisher Scientific; APC Mouse Anti-Human CD9, Clone HI9a, 312,108; BioLegend, Saa Diego, CA, USA; PE-Cy7 Mouse Anti-Human CD63, Clone H5C6, 561,982; BD Biosciences) and Western blot (anti-flotillin-1 1:10,000, ab41927; anti-CD9-1:1000 EPR23105-121, Abcam, Cambridge, UK), as already described [21]. In addition, the protein contamination (*i.e.,* lipoproteins) was also investigated by ELISA using Human ApoA1 and Human apoB Kits (Mabtech, Inc., Cincinnati, USA); specifically, in each plate, 100 µl of plasma (1:5000), isolated plasma EVs (1:2), and standard samples were run in duplicate and analyzed according to the manufacturer’s instructions. The EV-miRNome was profiled by microarray using SurePrint Human miRNA 8X60K (Agilent Technologies, Santa Clara, CA, USA; AMADID: 070156). Briefly, for microarray labeling, 6 µl of EV-RNA isolated from 1 mL of plasma sample and eluted in 19 µl were processed as already described [22]; for samples where the input plasma volume differed from 1 mL, the volume of EV-miRs used for microarray labeling was adjusted based on the initial plasma volume (Supplementary Table S1). The image processing with Feature Extraction *v.*9.5.3.1 (Agilent Technologies) and data preprocessing with LIMMA package for microarray, available within R statistical software background correction and between array normalization were carried out using the normexp method, with an offset = 20, and the scale method, respectively. Probes non-detected in more than 50% of the short survivors (SS, survival time < 9 months) and long survivors (LS, survival time ≥ 9 months) were filtered out. Then, replicated probes and replicated miRs were averaged.

### EV-miRNA validation

Based on the starting plasma volume (1–0.5 mL), 4–8 µl of EV-RNA were reverse-transcribed using the miRCURY LNA RT Kit (Qiagen, Supplementary Table S1). Then, 2–4 µl of diluted complementary DNA (cDNA, 1:20–30) were amplified using iTaq Univer SYBR Green Supermix (Bio-Rad, Hercules, CA, USA) with 1 µl of specific miR primers (Supplementary Table S2). The relative concentration was calculated as [2^-(EV-miR-Ct-Mean—UniSP6-Ct-Mean)] (Ct: threshold cycles).

### *PD-L1 gene absolute* quantification

The EV-PD-L1 mRNA copy number (CN/1 mL) was assessed by the QX200 droplet digital PCR (ddPCR) system (Bio-Rad). Briefly, 2 µl of EV-RNA were reverse-transcribed using SuperScript™ IV VILO™ Master Mix (Thermo Fisher Scientific). Then, 10 µl of cDNA were amplified using the ddPCR Supermix for Probes (No dUTP) (Bio-Rad) with the PD-L1 FAM-labeled assay (dHsaCPE5058502). Quantification was assessed using the QuantaSoft software (Bio-Rad) in bidimensional visualization, applying the threshold based on the negative template control signal. Samples with a droplet number < 10,000 were repeated.

### Functional enrichment and pathway analyses

The Kyoto Encyclopedia of Genes and Genomes (KEGG) pathway and gene ontology (GO) enrichments were performed by DIANA-miRPath v.3.0 [23] and MIcroRNAENrichmentTURnedNETwork (MIENTURNET) [24] (June 2022) free web tools. Outputs were selected based on enrichment False Discovery Rate < 0.05.

### Statistical analysis

To build the prognostic score, we used a 9-month cutoff based on the CheckMate 017 results [1] splitting the training set (*n* = 174) in SS and LS patients. Class comparison was performed using the R limma package between (i) SS (*n* = 96) versus LS (*n* = 78) and (ii) BR versus baseline (*n* = 54 SD/PR patients). To select EV-miRs associated with overall survival (OS), the patient cohort was randomly split 10 times into subgroups made up of 70% of the patients (125/174, with every patient selected at least once), maintaining the same proportion of SS and LS patients in each subset [25]. A penalized Cox regression model using the LASSO method was built on each subgroup using the *cv.glmnet* function of the glmnet R package, performing a tenfold cross-validation step to fit the model, and the EV-miRs with nonzero coefficients were retained. The EV-miRs retained by at least eight out of ten models were chosen for the validation step. In addition, the EV-miRs retained from five to seven models were further selected based on the model coefficients and available literature. Specifically, their potential role in cancer was assessed using PubMed and the following keywords: “miR-ID AND Cancer” or “miR-ID AND Lung cancer” (December 2021). Hence, for each miR, the impact was calculated by multiplying the number of papers by its regression coefficient; EV-miRs with absolute impact greater than 1 were selected for validation. Spearman correlation between microarray and qPCR data was tested using the *cor.test* function of the stats R package. The *coxph* function of the survival R package was applied on the samples with qPCR data available (*n* = 104) to perform univariable and multivariable Cox regression. Stepwise Cox regression was carried out to find the EV-miR combination (with or without clinical variables) with best prediction accuracy, using the *step* function of the stats R package. Collinearity between selected variables was assessed through the *vif* R function in the caret package. Risk scores were calculated by means of a weighted sum of the variables in the model, where the weights are the Cox regression coefficients (logHR). Predictor values were centered using their overall means. The best model was then tested on an independent cohort of patients with the *predict* R function. Kaplan–Meier curves and UNO’s area under the curve (AUC) were calculated to assess the performance of the models, using the *ggsurvplot* (survminer package) and *timeROC* R functions, respectively. To test the differences between time points (TPs) during therapy, Wilcoxon signed rank-sum test was performed, and the *pairwise.Wilcoxon.test* R function was used to obtain the p-values from the comparison between groups. P-values were adjusted following the Benjamini–Hochberg procedure.

## Results

### Study population

Two hundred and forty-five patients (174 training set and 71 test set) with advanced NSCLC were enrolled to receive nivolumab in the second-line setting (Table 1 and Supplementary Table S1). The mean age was 66.6 (range: 37–88). Most of the patients were male (70%) with a history of smoking habit (87%). Tumor histology was adenocarcinoma in 76%. At the time of data analysis, 212 patients (86%) had died. Median progression-free survival (PFS) and OS were 3 and 7.5 months, respectively. Notably, 56 patients experienced early death (ED), defined as an event that occurred before undergoing the first CT. Among the remaining 190 patients, 96 (50%) experienced PD as BR, while 41 patients (22%) achieved PR, 49 (26%) SD, and 4 (2%) did not undergo further assessments due to worsened clinical conditions. Overall, both training and test cohorts were similar in terms of clinical features, although slightly more patients of the training set (50% vs. 8.5% of the test set) received more than one-line therapy before nivolumab administration.

**Table 1:** Clinical and pathological characteristics of the training set and test set

Training set characteristics	*n*	*%*	Test set characteristics	*n*	*%*
*Patients*	174	–	*Patients*	71	–
*Age*			*Age*		
< 70	104	59.8	< 70	37	52.1
≥ 70	70	40.2	≥ 70	34	47.9
*Gender*			*Gender*		
Male	123	70.7	Male	48	67.6
Female	51	29.3	Female	23	32.4
*Histotype*			*Histotype*		
No squamous cell carcinoma	134	77.0	No squamous cell carcinoma	53	74.6
Squamous cell carcinoma	40	23.0	Squamous cell carcinoma	18	25.4
*ECOG PS*			*ECOG PS*		
1	148	85.1	1	58	81.7
2	26	14.9	2	8	11.3
Not available	0	0.0	Not available	5	7.0
*Smoking status*			*Smoking status*		
Never	15	8.6	Never	12	16.9
Former and current smoker	159	91.4	Former and current smoker	52	73.2
N.A	0	0.0	N.A	7	9.9
*Metastasis*			*Metastasis*		
Only brain	21	12.1	Only brain	13	18.1
Only liver	38	21.8	Only liver	11	15.3
Brain and liver	7	4.0	Brain and liver	2	2.8
Other metastasis sites	122	70.1	Other metastasis sites	49	69.0
*Prior lines of treatment*			*Prior lines of treatment*		
1	87	50.0	1	60	84.5
> 1	87	50.0	> 1	6	8.5
N.A	0	0.0	N.A	5	7.0

*NA* not available

### Construction and validation of a prognostic EV-miR and clinical data score

**Fig. 2:**
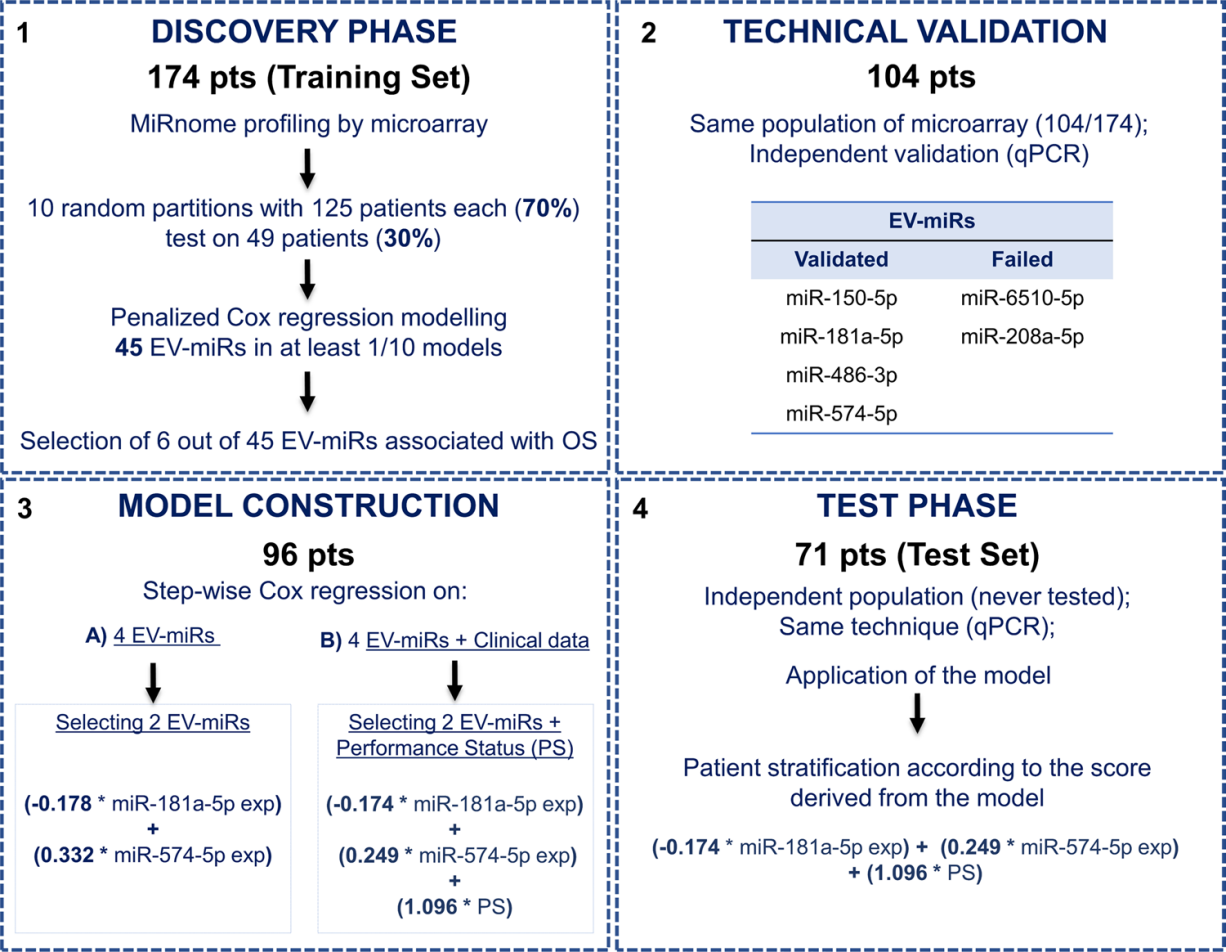
Flowchart of the four steps to build the prognostic score. *Exp* expression level and *Pts* patients

The prognostic score was obtained through four consecutive steps (Fig. 2).

#### Discovery phase

Initially, the purity and the EV enrichment were assessed on a pool of plasma from 10 healthy individuals (HIs: 5 males + 5 females). Notably, we found a high enrichment of small and large EVs (mean: 196 nm, 87–32 nm) with a weak contamination of albumin/IgG (~ 1%) and almost total absence of Apo-B100 lipoprotein (0.003%) and in the isolated EVs compared the non-processed plasma (Supplementary Fig. 1A and B). To further verify the integrity of miRs within the EVs, we successfully detected and quantified EV-miRs in all samples (mean 1.7 pg/µl, ranging from 0.1 to 5.2). Notably, we did not observe any correlation between the amount of EV-miRs and the time of freezing of the samples (Spearman correlation coefficient = − 0.27, *p*-value = 0.13; Supplementary Fig. 2A). The whole EV-miRNome of 174 patients reported a median number of 375 detected EV-miRs (range: 100–785). When looking at the difference between SS and LS, the median number of detected EV-miRs was significantly higher in the first group (390 in SS versus 347 in LS; *p*-value = 0.04). For the prognostic EV-miR-based score, the training cohort was randomly split 10 times into subgroups made up of 70% of the patients, with every patient selected at least once (Fig. 2A and Supplementary Table S3) [25], and a penalized Cox regression model was built on each subgroup identifying 45 EV-miRs retained by at least one model. Among these, we selected six EV-miRs as follows: four (miR-150-5p, miR-208a-5p, miR-6510-5p, and miR-574-5p) retained from eight out of ten models and two further EV-miRs (miR-181a-5p and miR-486-3p) with absolute impact > 1 (Supplementary Table S3). We have also included some confounding factors such as histotype, number of treatment lines, sex, and performance status along with the EV-miRs into the LASSO variable selection process. Notably, none of these variables was selected by the LASSO shrinkage method, indicating their minimal impact on the prognosis of our cohort, except for performance status which was retained from more than 8 models.

#### Technical validation

**Fig. 3:**
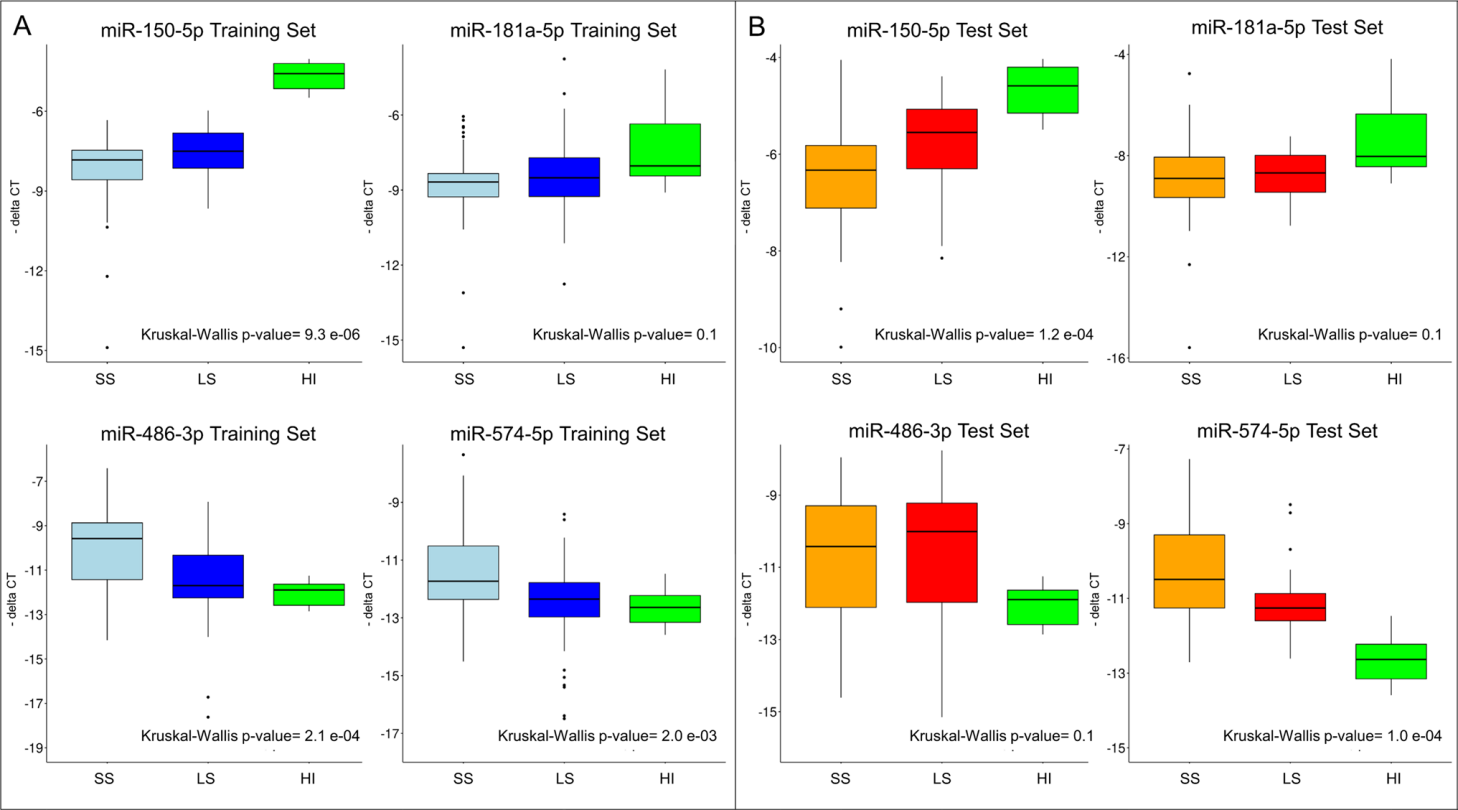
Box-plots of EV-miR expression assessed by qPCR on cancer patients and healthy controls. **A** Box-plots of the four EV-miRs in the training set: 55 short survivors (SS: OS < 9 months; light blues); 49 long survivors (LS: OS ≥ 9 months; blues); and 24 healthy individuals mixed in six pools (HI, green). **B** Box-plots of the four EV-mRs in the test set: 42 SS (orange); 29 LS (red); and 24 HI (green)

The expression of the six selected EV-miRs was assessed by qPCR on a subset of 104/174 samples with available plasma (Fig. 2B and Supplementary Table S4). For the qPCR normalization step, we initially selected miR-1228-3p as the most stable EV-miR revealed by microarray analysis. Even if it had been previously proposed as an EV-miR reference [26], qPCR did not detect its expression in any samples. Hence, we decided to normalize qPCR data using an internal miR Spike-in Control (UniSp6) added to our samples before RNA isolation. Two EV-miRs were not validated, the first one (EV-miR-6510-5p) failed qPCR amplification; on the contrary, EV-miR-208a-5p was successfully amplified, although its expression levels across the patient samples showed an inverse correlation between qPCR and microarray data (Supplementary Fig. S2B). The other four EV-miRs showed a positive correlation between qPCR and microarray data (*p*-value < 0.03). The expression levels of these four EV-miRs were also evaluated in a cohort of 24 healthy individuals (HIs) grouped into six pools, each containing three males and one female. Sex distribution between the two groups was similar (75% males for HIs *vs* 70% males for patients, Chi-square test, *p*-value = 0.52). Although age and EV-miR content were significantly lower in controls *vs* patients (Wilcoxon test, *p*-values equal to 3.4e−07 and 0.0056, respectively), the expression analysis generally confirmed the dysregulation of EV-miRs in SS and LS NSCLC patients compared to controls (Fig. 3). In particular, the expression levels of four EV-miRs in HIs exhibited different trends both higher (miR-150-5p and miR-181a-5p) and lower (miR-574-5p and miR-486-3p) values than the patients, with more pronounced differences observed in the SS individuals suggesting a potential association between EV-miR expression levels and a poorer clinical outcome. Then, the four EV-miRs were used for the model construction.

#### Model construction

**Fig. 4:**
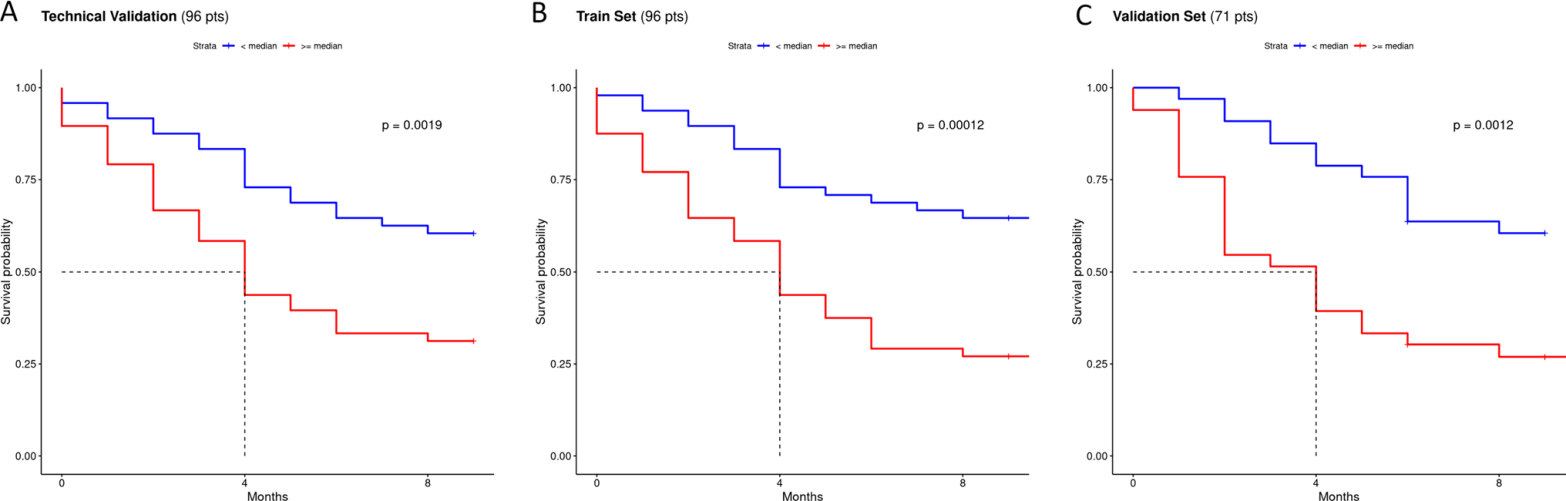
Prognostic score performance in the training and test sets. **A** Kaplan–Meier curves obtained by stratifying the training set with all the validated EV-miR (*N* = 96) data available, according to the median of the EV-miR-based prognostic score. **B** Kaplan–Meier curves obtained by stratifying the same patients according to the median of the prognostic score including also the PS. **C** Kaplan–Meier curves obtained by stratifying the test set (*N* = 71) according to the median of the prognostic score obtained by combining EV-miR expression plus the PS. Median OS in high-risk patients (red) was equal to 4 months, while low-risk subjects (blue) did not reach the median within 9 months, both in the training sets (panels A-B) and in the test set (panel C)

Ninety-six of 104 patients with qPCR data for all four EV-miRs and clinical data available were used for score construction by stepwise Cox regression model (Fig. 2C). Then, two models were built: one with EV-miRs only and another one including clinical variables. In both cases, only EV-miR-181a-5p and EV-miR-574-5p were selected but the best combination to predict OS was the one with the two EV-miRs plus performance status (PS). No collinearity between the three predictors was observed, as confirmed by the very low variance inflation factors (EV-miR-574-5p VIF = 1.082; EV-miR-181a-5p VIF = 1.001; and PS VIF = 1.0761). The combined prognostic score showed a good prediction of survival at 9 months (time-dependent Uno’s AUC = 0.76). High-risk subjects showed a median OS of 4 months compared with low-risk patients who did not reach the median within 9 months, both in the model with two EV-miRs (Fig. 4A; log-rank test *p*-value = 0.0019) and in the combined model that also included PS (Fig. 4B; log-rank test *p*-value = 0.00012). The same model gives good prognostic stratification of patients when we consider follow-up longer than 9 months, not only for OS but also for PFS status (Supplementary Fig. S3).

### Test on an independent cohort

Finally, four EV-miRs were tested by qPCR on an independent set of patients never tested (*n* = 71) confirming similar trends in SS, LS, and HI (Fig. 2 and Supplementary Table S5). The combined score integrating both EV-miRs and PS [(− 0.174 * miR-181a-5p exp) + (0.249 * miR-574-5p exp) + (1.096 * PS)] was obtained by multiplying specific coefficients for the value of each covariate. For the two EV-miRs, their logarithmic expression values (exp = delta Ct (CtEV-miR minus CtUniSP6)) were used, whereas for the PS variable, a binary variable was employed, assigning a value equal to “0” for PS = 0 and “1” for PS = 1, 2. Stratification of patients according to this score yielded a good separation (Fig. 4C; log-rank test *p*-value = 0.0012), with high-risk patients having a median OS of about 4 months; in contrast, low-risk patients did not reach the median within 9 months after starting nivolumab. Notably, the combined score reported a similar predictive ability for survival in the test set (Uno’s AUC = 0.77).

### EV-PD-L1 mRNA copy number evaluation

**Fig. 5:**
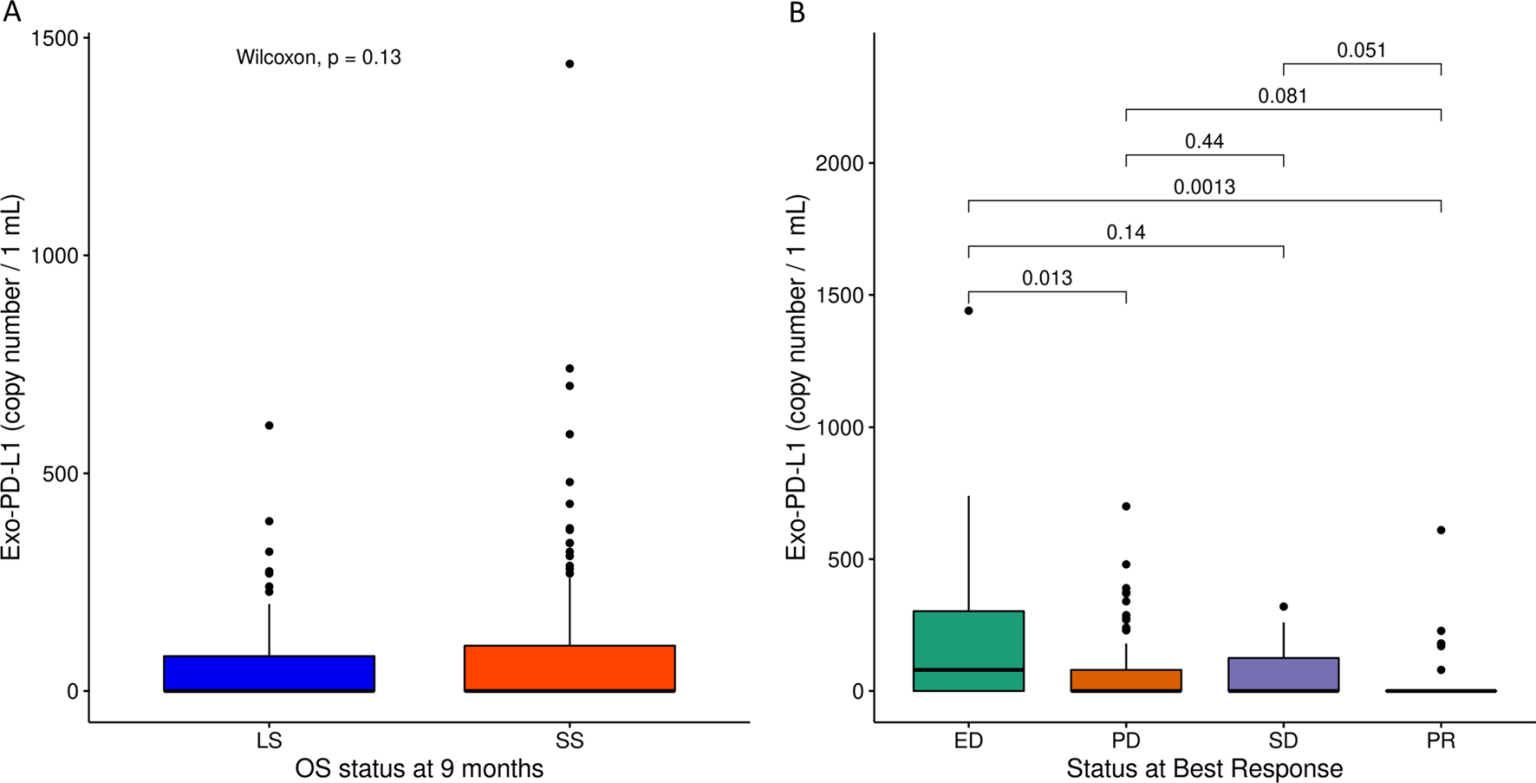
Box-plots of the EV-PD-L1 mRNA copy number (**A** Box-plots of the EV-PD-L1 mRNA in long survivors (LS; OS ≥ 9 months; 90 patients, pts; red) and short survivors (SS OS < 9 months; 95 pts; light blue). **B** Box-plots of the EV-PD-L1 in early death (ED; 26 pts; red), progression disease (PD; 90 pts; green), stable disease (SD; 27 pts; light blue), and partial response (PR; 34 pts; violet) patients. *p-value < 0.05

PD-L1 EV-mRNA copy number (CN/1 mL) on 185 patients showed an average of 81.7 (0.0–1440). When we estimated its prognostic effect, we found a generally higher mean value in SS (107.1) than in LS (54.7) patients (Fig. 5A). Notably, the highest average EV-PD-L1 CN was observed in ED patients (202.8) compared to PD (68.5) or SD (68.7), while few copies (42.6) were found in PR (Fig. 5B). However, when we tried to incorporate the copy number of the EV-PD-L1 gene into our model to test its impact on the prognostic score, we did not achieve statistical significance when we added it as a covariate in the multivariable Cox regression model (*p*-value = 0.296). We also used the stepwise algorithm for variable selection in a multivariable Cox regression model, but EV-PD-L1 copy number was not selected.

### Modulation of EV-miRs during therapy in patients with a disease control

Finally, to investigate the potential role of EV-miRs in modulating the immune system during treatment, we profiled the EV-miRNome in a subset of disease-controlled patients (31 stable disease, SD and 23 partial response, PR) and compared expression levels at BR versus baseline. The analysis identified 11 deregulated EV-miRs (i.e., eight miRs in SD + PR and three miRs in PR only) that were then validated by qPCR on 47 pts, 27 SD and 20 PR, confirming nine significantly downmodulated EV-miRs (Table 2 and Supplementary Table S6; Pearson correlation coefficient = 0.89, *p*-value = 0.005).

**Table 2:** List of EV-miRs differentially expressed between BR time versus baseline

EV-miRs	BR	logFC (microarray)	*p*-value (microarray)	logFC (qPCR)	*p*-value (qPCR)
hsa-miR-7977	SD+PR	− 0.58	1.60E−05*	− 2.16	1.58E−03*
hsa-miR-7975	SD+PR	− 0.56	3.15E−05*	− 2.08	5.59E−06*
hsa-miR-142-3p	SD+PR	− 0.56	3.07E−03*	− 2.02	2.30E−06*
hsa-miR-19a-3p	SD+PR	− 0.53	2.72E−04*	− 1.92	3.54E−04*
hsa-miR-20a-5p	SD+PR	− 0.51	1.09E−03*	− 1.66	5.55E−04*
hsa-miR-5100	SD+PR	− 0.48	7.76E−05*	− 1.30	2.52E−04*
hsa-miR-7641	SD+PR	0.45	2.54E−03*	− 0.08	0.8455
hsa-miR-3610	SD+PR	0.50	1.62E−04*	*Failed*	–
hsa-miR-1260b	PR	− 0.52	5.91E−04*	− 1.72	1.48E−03*
hsa-miR-1260a	PR	− 0.56	1.63E−03*	− 1.84	1.78E−04*
hsa-miR-26a-5p	PR	− 0.52	3.16E−02*	− 1.52	8.91E−03*

The table reports the logarithmic fold changes (logFC) and *p*-values for 11 EV-miRs obtained by microarray (*N* = 54) and qPCR (*N* = 47) (*p-value < 0.05)

**Fig. 6:**
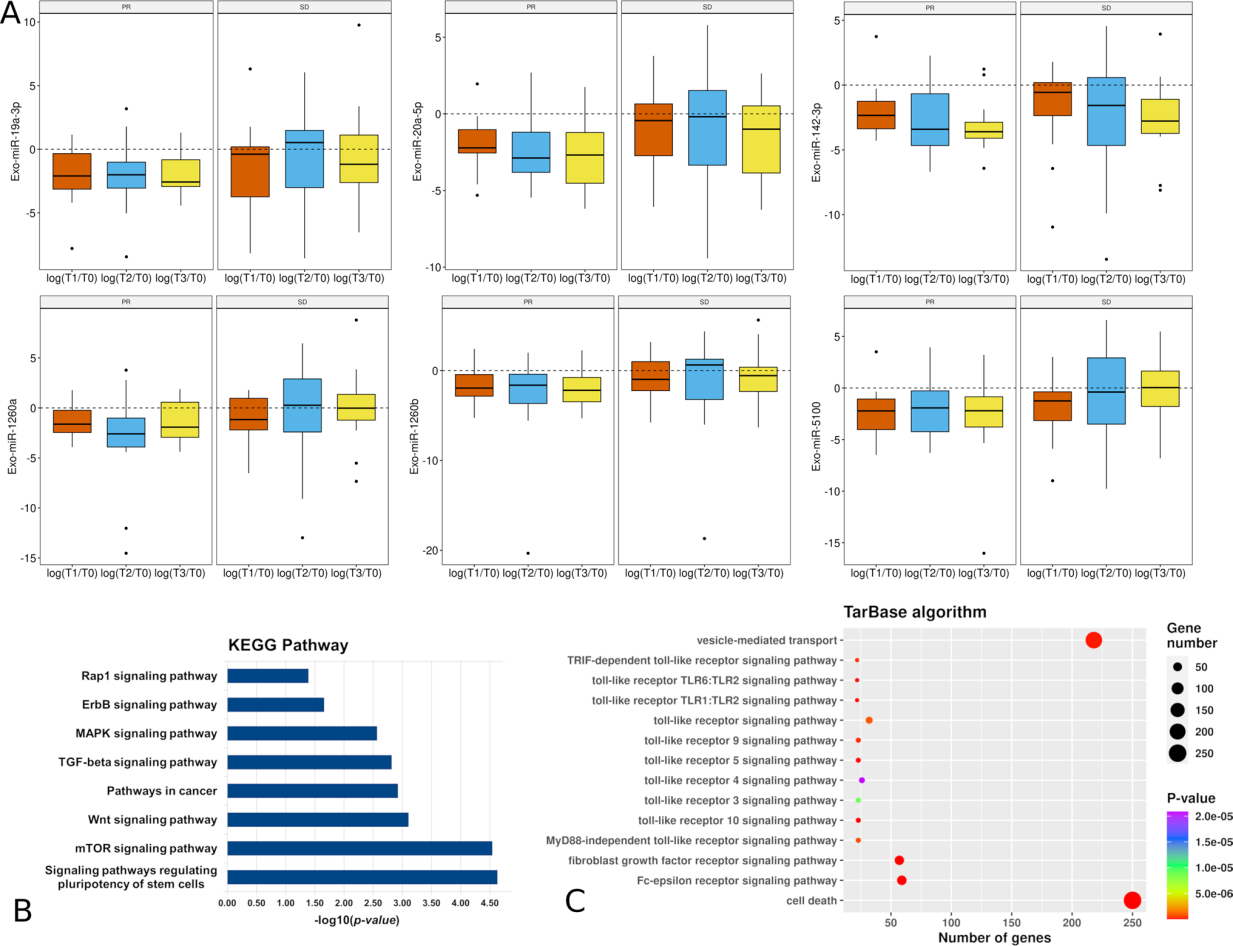
EV-miR deregulation during therapy and pathway analysis based on the EV-miR targets. **A** Box-plots of qPCR expression log fold-change at each time point (T1: 1st CT scan; T2: 2nd CT scan; and T3: 3rd CT scan) versus baseline (T0), for the six downregulated EV-miRs in PR (left panels) and SD patients (right panels). **B** List of the most relevant KEGG pathways (by Diana tool) related to the predicted targets of the six downmodulated EV-miRs. **C** Bubble plot of the most relevant Gene Ontology biological processes related to the biologically validated targets of the six downmodulated EV-miRs

Then, the downregulated EV-miRs were monitored at three consecutive CT-scans (T1: 2 months; T2: 4 months; and T3: 6 months) (Supplementary Table S7). Notably, 6/9 EV-miRs (miR19a-3p, miR-20a-5p, miR-142-3p, EV-miR-1260a, miR-1260b, and miR-5100) were significantly downmodulated at all time points in PR patients, whereas no miR was deregulated in SD patients (Fig. 6A and Supplementary Table S8).

Enrichment analysis of the predicted targets of the six silenced EV-miRs identified signaling pathways previously described in the response to ICI, such as stem cell pluripotency, an approach exploited to enhance the anti-tumor response [27] (Fig. 6B; Supplementary Table S9A; and Supplementary Fig. S4). Gene Ontology analysis showed a significant enrichment of toll-like receptor (TLR) signaling pathways (Fig. 2C; Supplementary Fig. S5; and Supplementary Table S9B).

## Discussion

Here, we performed an extensive EV-miRNome profiling in a large cohort of advanced NSCLC patients who received nivolumab after failure of previous therapies. The analysis showed a higher enrichment of EV-miRs in the plasma of patients with shorter OS (*p*-value = 0.04); this result is not surprising; indeed, a higher EV-miR enrichment has already been positively associated with cancer progression [28]. Prognostic EV-miRs were initially obtained by applying a penalized Cox regression model on microarray data identifying six EV-miRs able to discriminate between short- and long-term survivors. Subsequent technical validation using qPCR confirmed the prognostic potential of four out of the six EV-miRs (i.e., EV-miR-150-5p, EV-miR-181a-5p, EV-miR-486-3p, and EV-miR-574-5p). The discordant results between the microarray and qPCR data could be linked to the limitations of qPCR for low-abundance EV-miRs resulting in decreased sensitivity [29]. In addition, the short length of miR and their high sequence homology could pose a challenge in differentiating closely related miRs [30], although we cannot exclude a false-positive result from the microarray [31]. Notably, all four validated EV-miRs have been previously described in lung carcinogenesis [32–35] as well as in the EV-trapped forms [36–40]. In particular, EV-miR-150-5p has been defined as an immune modulator [41] that targets regulator genes (i.e., IL-10 and PIM1) of the myeloid-derived suppressor cells (MDSC) which are potent suppressors of immune responses mediated by T lymphocytes and NK cells [42]. Indeed, miR-150-5p reported a negative coefficient in Cox regression analysis, confirming its immune modulator role. A combination of EV-miR-181a-5p and EV-miR-574-5p together with performance status resulted in the best prognostic model associated with OS in the training set, and the ability to predict survival was confirmed in an independent cohort of 71 patients with similar characteristics. Interestingly, miR-181a-5p has been linked to T-cell activation [43–45], as well as interferon-gamma (IFNG) overexpression and natural killer (NK) cell maturation [46, 47]. In addition, miR-181a has also been demonstrated to inhibit NSCLC cell lines [48], and the circulating form has been described as a marker for diagnosis and good prognosis in NSCLC [40–43, 48, 49], also confirmed by its negative Cox regression coefficient. An increasing number of studies have also demonstrated that miR-574-5p overexpression correlates with lung cancer progression and metastasis [50–52], whereas its soluble form has been described as a promising marker for patient stratification with NSCLC [39, 51, 53, 54]. The positive Cox regression coefficient of miR-574-5p in our model supports its association with poor prognosis/response to ICIs. Overall, our findings support the hypothesis that evaluation of these EV-miRs in the plasma of patients receiving ICIs may help in predicting response to immunotherapy.

In addition, we also confirmed that higher EV-PD-L1 CN was linked to worse outcomes, particularly in patients with ED, but the addition of EV-PD-L1 CN to the EV-miR-based score did not improve the significance of the prognostic prediction model. Currently, growing evidence demonstrates that EV-mRNA molecules, contrarily to small RNAs, can be found either as functionally mRNA source or non-functional fragmented form [55]. Hence, we can speculate that EV-PD-L1 mRNA may primarily be a non-functional degradation product linked to an aggressive disease, rather than a source for protein translation in recipient cells.

Concomitantly, we investigated the EV-miR changes at BR in patients experiencing disease control disclosing a significant downmodulation of nine EV-miRs at BR *vs* baseline. Notably, in patients experiencing a PR, six out of nine EV-miRs (*i.e.,* miR19a-3p, miR-20a-5p, miR-142-3p, miR-1260a, miR-1260b, and miR-5100) were already downregulated at the time of the first CT scan evaluation and remained silent over 6 months of therapy. Currently, accumulating evidence has demonstrated an oncogenic role of these circulating miRs in lung cancer progression [56–61]. In addition, the previous studies have shown that EV-miR-20-5p is a negative T-cell regulator [62], and that miR-19a-3p is able to target major histocompatibility complex (MHC) class I genes [63]. In particular, MHC genes are involved in the adaptive immune response, and their downmodulation has been described as a mechanism of resistance to ICI [64]. Concerning that point, Jiang and colleagues recently demonstrated that EVs deriving from pro-inflammatory macrophages (i.e., M1 phenotype) were able to silent miR-19a-3p through a long non-coding RNA (lncRNA: HOXA transcript at the distal tip). This leads to an upregulation of the toll-like receptor 5 (TLR) signaling which, in turn, activates the polarization of the circulating monocytes into M1 macrophages [65]. Similarly, functional enrichment analysis on the predicted targets of the EV-miR downmodulated in responding patients showed an over-representation of TLR signaling (e.g., 1, 2, 3, 4, 5, 6, 9, and 10). TLRs play a dual regulatory role in cancer with both anti-tumor and pro-tumor effects, depending on their class and cancer type [66]. In particular, TLR3 expression on NSCLC cells has been described associated with apoptosis activation, induced by caspase-3. Moreover, TLR3-mediated apoptosis also increased the activation of immune response in NSCLC through CD103 + dendritic cells [67]. In this regard, TLR3 agonists have been proposed in clinical use as adjuvant to overcome the resistance to ICIs [68]. Enrichment analysis also showed an activation of cellular pathways such as induction of pluripotency of stem cells (PSCs), a reprogramming phenomenon from a somatic cell. To date, numerous in vivo studies have shown that induced PSCs might be exploited to improve the anti-tumor response, for example, by generating T lymphocytes with a wide variation of T-cell receptor rearrangement patterns [27]. More recently, Cichocki et al., reported that the activation of induced NK cells, derived from PSCs, may overcome the resistance of PD-1 blockade, by recruiting T cells with increased production of inflammatory cytokines [69]. Altogether these results support a role in the activation of immune pathways, strengthening the immune system against cancer cells and inducing tumor suppression.

Despite several breakthroughs in our study, some limitations should be considered. Firstly, a relevant proportion of the enrolled patients experienced PD at the first response assessment. Furthermore, a non-negligible fraction of patients experienced worsened clinical conditions and eventually died before undergoing the first response assessment. While this occurrence might appear unexpected, it depends on the fact that nivolumab was initially available through an expanded access program, which allowed the prescription of the agent to heavily pre-treated patients with increased tumor burden and more compromised performance status which limited life expectancy. The current trend in clinical practice includes the use of ICI in earlier settings compared to our study population; thus, our score should be confirmed in treatment-naïve cohorts undergoing immunotherapy. Another critical point of our study is represented by the lack of validation involving multiple laboratories, encompassing the whole experimental workflow, from EV-miR isolation to qPCR validation and data analysis.

## Conclusions

To the best of our knowledge, our study represents the largest EV-miR analysis, providing a robust and reliable prognostic score, based on the expression of two EV-miRs (EV-miR-181a-5p and EV-miR-574-5p) and clinical data (*i.e.,* PS) able to identify NSCLC patients who could benefit from immunotherapy. EV-PD-L1 CN, on the other hand, although associated with progressive disease, did not improve the EV-miR score, leading to speculation that it can be a degradation product. In addition, the targets of the six EV-miRs downregulated at the time of BR are associated with the modulation of immune system cells toward a stem phenotype that might generate, for instance, lymphocytes with a wide variation of T-cell receptor rearrangement patterns, a hypothesis also supported by TLR enrichment in responders. Our findings, if validated in independent cohorts, provide the basis for a better patient selection in line with immunotherapy personalization and open new avenues for the development of therapeutic strategies to improve response to nivolumab.

## Supplementary Information

Below is the link to the electronic supplementary material.

Supplementary file1 (PDF 820 KB)

Supplementary file2 (XLSX 1947 KB)

## Data Availability

All data generated during this study are included in this published article and its supplementary information files, whereas the datasets from microarray are available in Gene Expression Omnibus repository [http://www.ncbi.nlm.nih.gov/geo/, ID: GSE207715, released at the time of publication].

## Abbreviations

AUC: Area under the curve
BR: Best response
CN: Copy number
CT scan: Computed tomography scan
ddPCR: Droplet digital PCR
ED: Early death
EV-miRs: EV microRNAs
EV-PD-L1: EV-PD-L1
GO: Gene ontology
ICI: Immune checkpoint inhibitor
KEGG: Kyoto Encyclopedia of Genes and Genomes
LS: Long survivors
NSCLC: Non-small cell lung cancer
OS: Overall survival
PD: Disease progression
PD-1: Programmed cell death-1
PD-L1: Programmed cell death-1 ligand
PFS: Progression-free survival
PR: Partial response
qPCR: Quantitative PCR
SD: Stable disease
SS: Short survivors
TLR: Toll-like receptor
TPs: Time points
