# Supplementary material for: Extracellular vesicles miR-574-5p and miR-181a-5p as prognostic markers in NSCLC patients treated with nivolumab
Source: Clin Exp Med. 2024 Aug 6;24(1):182. doi: 10.1007/s10238-024-01427-8 (PMC11303437; doi:10.1007/s10238-024-01427-8)
Supplement: Supplementary file 1 — Supplementary file1 (PDF 820 KB) [file 10238_2024_1427_MOESM1_ESM.pdf]

Supplementary Figures

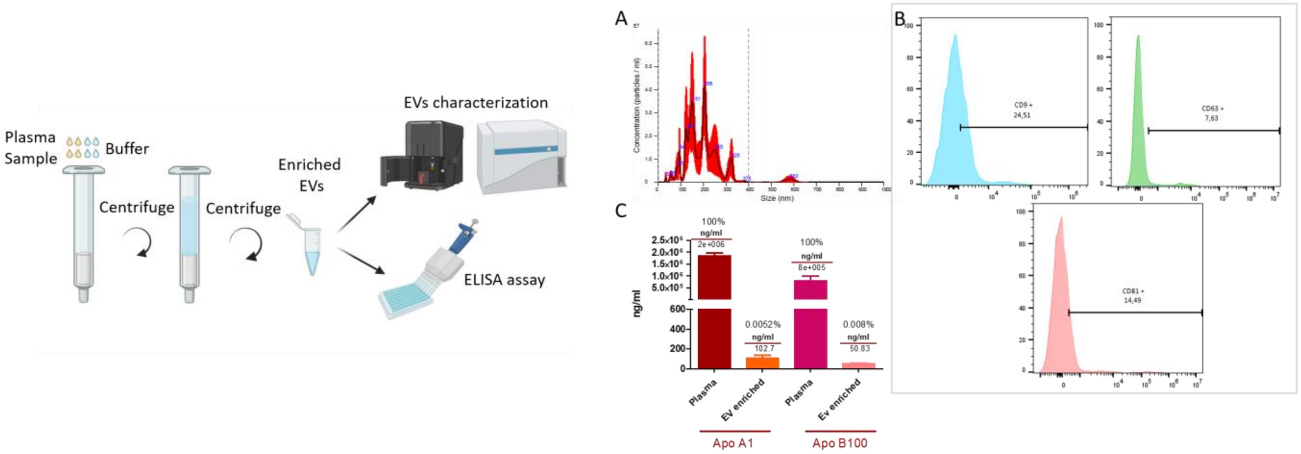

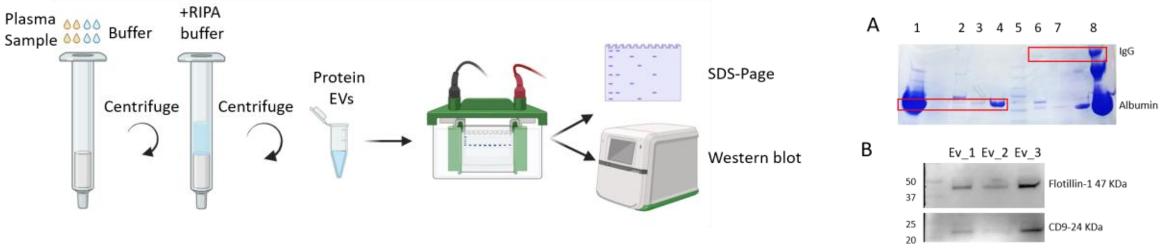

Supplementary Figure S1. EV Characterization

Upper Panel: EVs were isolated by 500 µl of plasma from a pool of 10 healthy individuals (HIs), using the membrane affinity column of the Exo-RNeasy Midi kit (Qiagen, Hilden, Germany) according to the manufacturer's instructions and stopping the protocol before the EV lysis. The collected EVs were then characterized by Nanoparticle Tracking Analysis (NTA), non-conventional flow cytometry, and enzyme linked immunosorbent assay (ELISA). **A)** NTA analysis showed a heterogeneous EV population of 3.50e+09 particles/mL; **B)**; **C)** ELISA assays of ApoA1 and ApoB100 proteins showed a strong decrease of the lipoprotein contamination in the isolated EVs (0.005% and 0.008%) compared to the non-processed plasma.

Lower Panel: HI pool EVs collected in the affinity column (EV-RNeasy Midi kit) were lysed in 110 µl of radioimmunoprecipitation assay buffer (RIPA) and analyzed by sodium-dodecyl-dulfate-polyacrylamid gel electrophoresis (SDS-Page) and western blot assays. **A)** SDS-Page in both reduced (lanes: 1-4) and non-reduced (lanes: 6-8) conditions showed significant lower levels of protein contaminations in the isolated EVs (0.2% albumin, 0.1% IgG) compared to the non-processed plasma. *Lanes*: "1, 10": 10 µg non-processed plasma; "2, 6": 10 µg Albumin/IgG depleted plasma (negative control); "3, 7": 50 µg isolated EVs; "4": 10 µg bovine serum albumin (positive control); "5": Precision Plus Dual Xtra standard. **B)** Western blot (electrophoresis at 120 V for 90 min; transfer with turbo blot transfer system at 2.5 A for 10 min) was performed with different EV total protein concentrations (20-50 µg), confirming the presence of CD9 and Flotillin-1 positive EVs. (Created with Biorender.com)

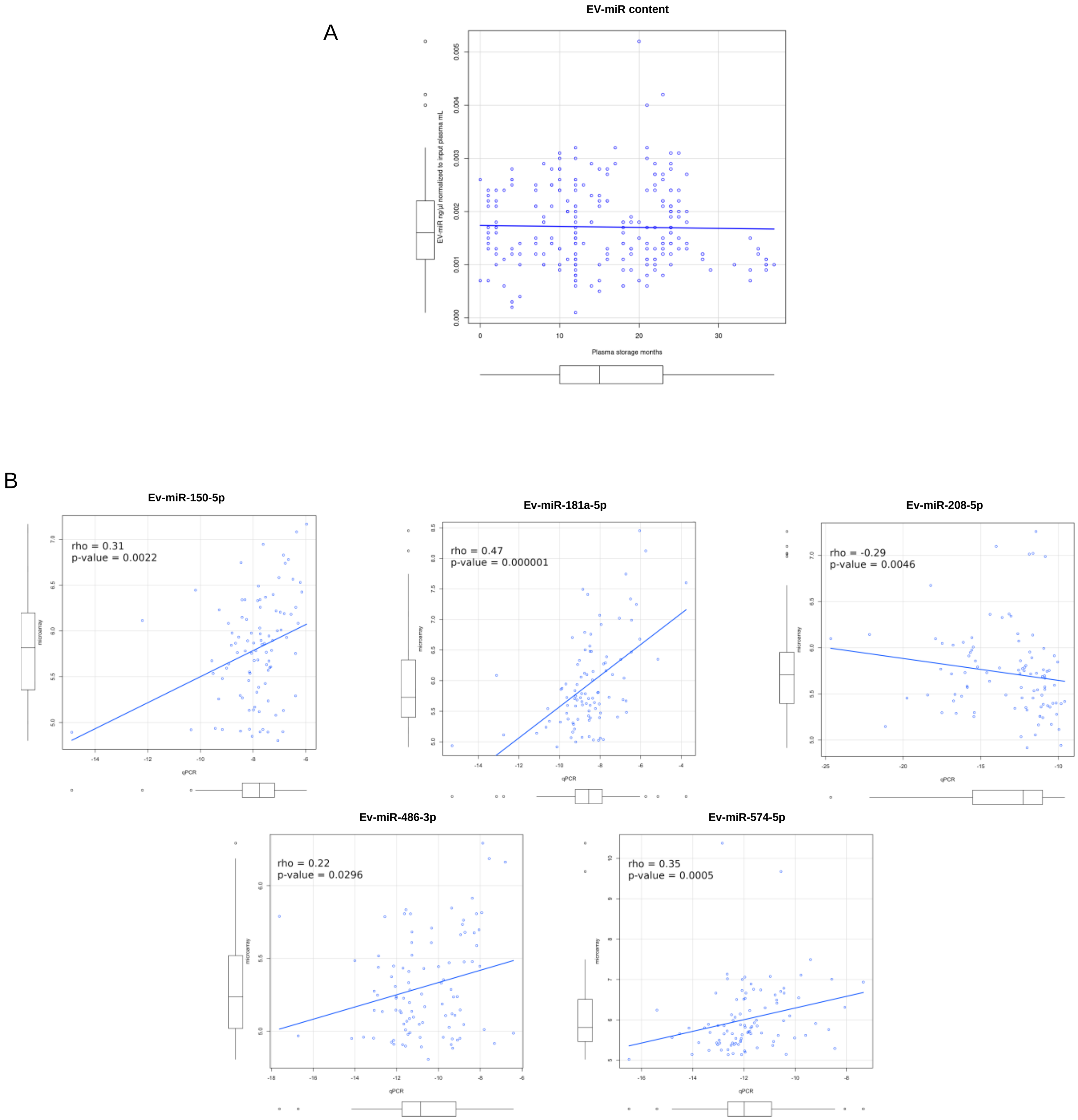

Supplementary Figure S2: **A.** Scatter plot of the relationship between EV-miR concentration (ng/µl by Qubit normalized to the volume (mL) of the input plasma sample) and storage time of plasma samples at 80°C (months). **B**. Scatter plots of EV-miR expression values comparing microarray and qPCR data.

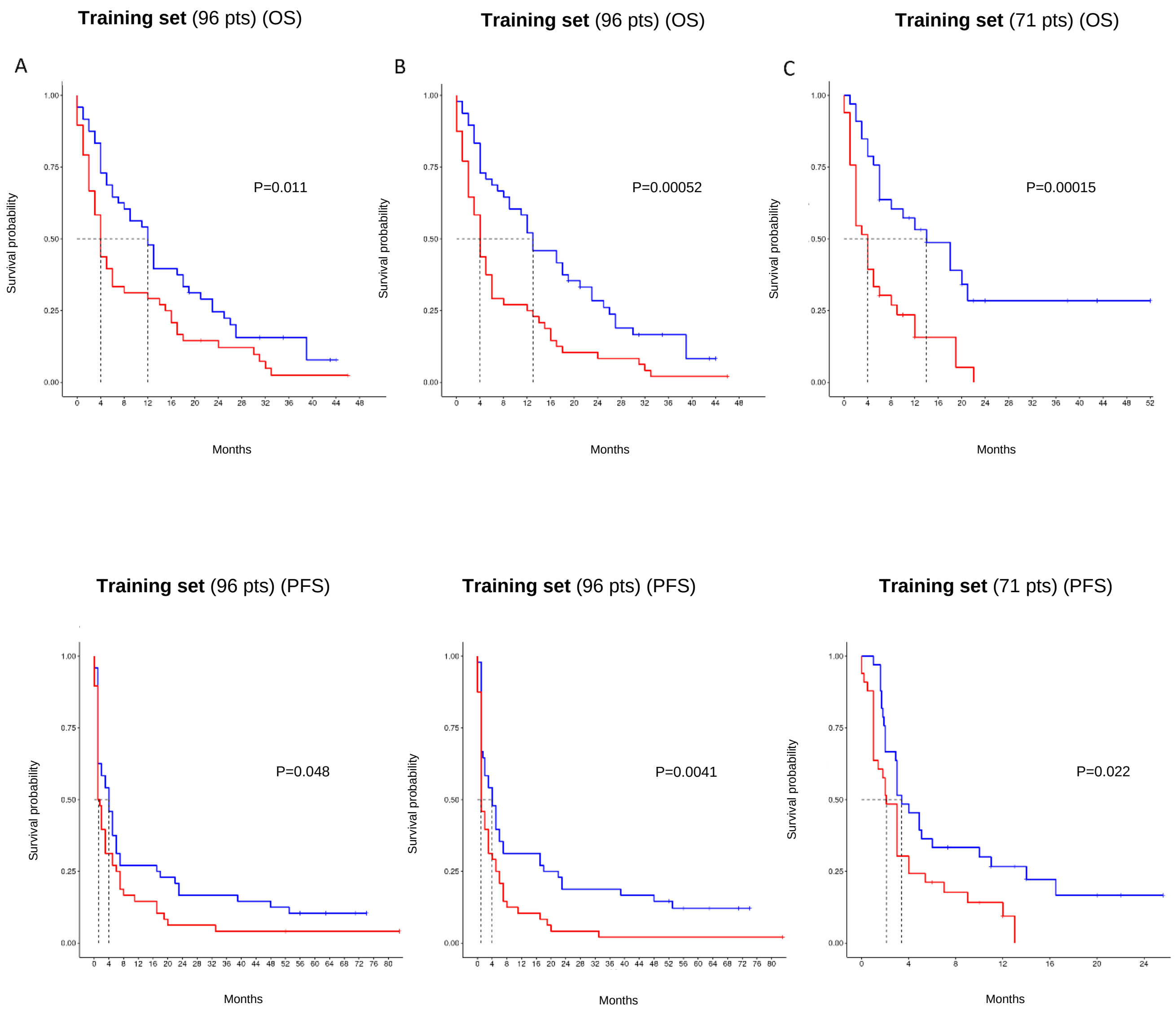

Supplementary Figure S2: **Prognostic score performance in the training and test sets with follow-up longer than 9 months.** (**A-D**) Kaplan-Meier curves obtained by stratifying the training set with the validated EV-miR (N=96) data available, according to the median of the EV-miR-based prognostic score. (**B-E**) Kaplan-Meier curves obtained by stratifying the same patients (N=96) according to the median of the prognostic score including also the PS. (**C-F**) Kaplan-Meier curves obtained by stratifying the test set (N=71) according to the median of the prognostic score obtained by combining EV-miR expression plus the PS. In panels A, B, and C the OS was considered while in panels C, D, and E the PFS was shown.

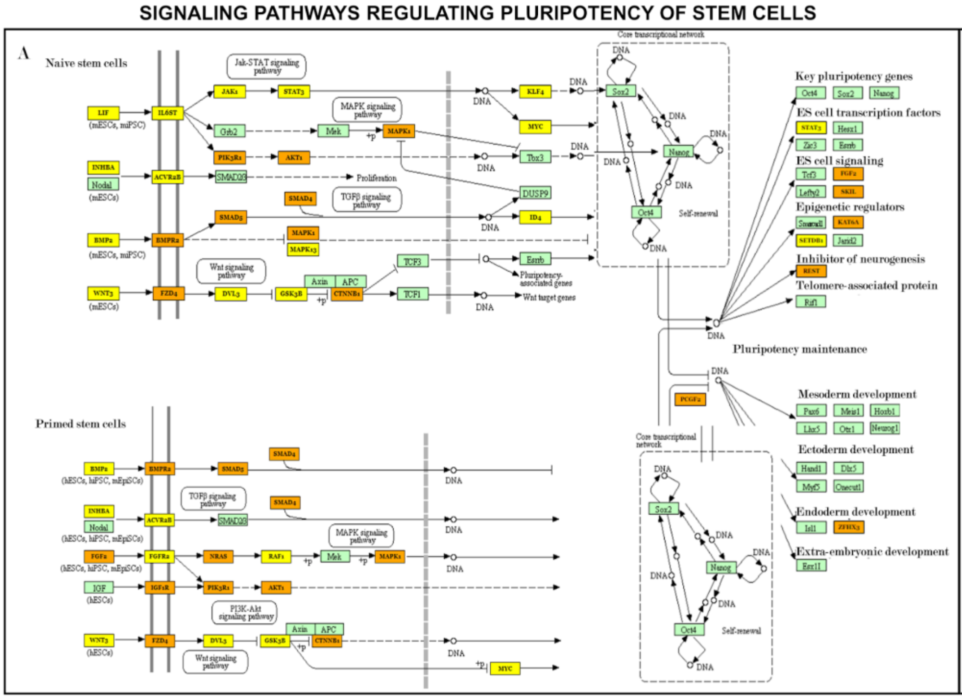

B

miRs	Genes
miR-19a-3p	GSK3B, FZD5, **NRAS**, INHBB, PIK3CB, **REST**, **PIK3R5**, MAPK14, **FZD6**, RAF1, INHBA, WNT3, **IGF1R**, **ZFHX3**, ID4, PCGF5, LIF, PIK3R3, **SMAD4**, **AKT1**, LIFR, **SKIL**, **SMAD5**, **PIK3R1**, **FGF2**, BMP2, FGFR2, **MAPK1**, **BMPR2**, **PCGF2**
miR-20a-5p	DVL3, STAT3, **KAT6A**, **NRAS**, **REST**, ACVR1B, **FZD6**, **IGF1R**, **ZFHX3**, FZD3, RIF1, **SMAD4**, **AKT1**, MYC, **SKIL**, **SMAD5**, **PIK3R1**, **FGF2**, IL6ST, **MAPK1**, JAK1, **BMPR2**, **PCGF2**
miR-142-3p	FZD7, **KAT6A**, PIK3R2, **PIK3R5**, MAPK13, AKT2, PIK3CD, **SMAD4**, **CTNNB1**, JAK3, PCGF3, KLF4, **BMPR2**
miR-1260a	**FZD8**, ACVR2B, **FZD4**, **CTNNB1**
miR-1260b	**FZD8**, SETDB1, **FZD4**, **SMAD4**, **CTNNB1**
miR-5100	**CTNNB1**

Supplementary Figure S4. (A) Schematic representation of the Signaling Pathways Regulating Pluripotency of Stem Cells by DIANA-miRPath *v*.3.0 (TarBase algorithm) using the list of the six EV-miRs silenced (down-modulated?) during the therapy in the responding patients. The green, yellow and orange boxes indicate no targeted gene, targeted gene contained in a list, targeted gene contained in more than one list, respectively. **(B)** For each miR, the targeted genes are listed. The gene shared between multiple miRs is reported in bold.

Abbreviations: mESCs: Mouse ES cells; miPSCs: Mouse iPS cells; hESCs: Human ES cells; hiPSCs: Human iPS cells; mEpiSCs: Mouse EpiS cells.

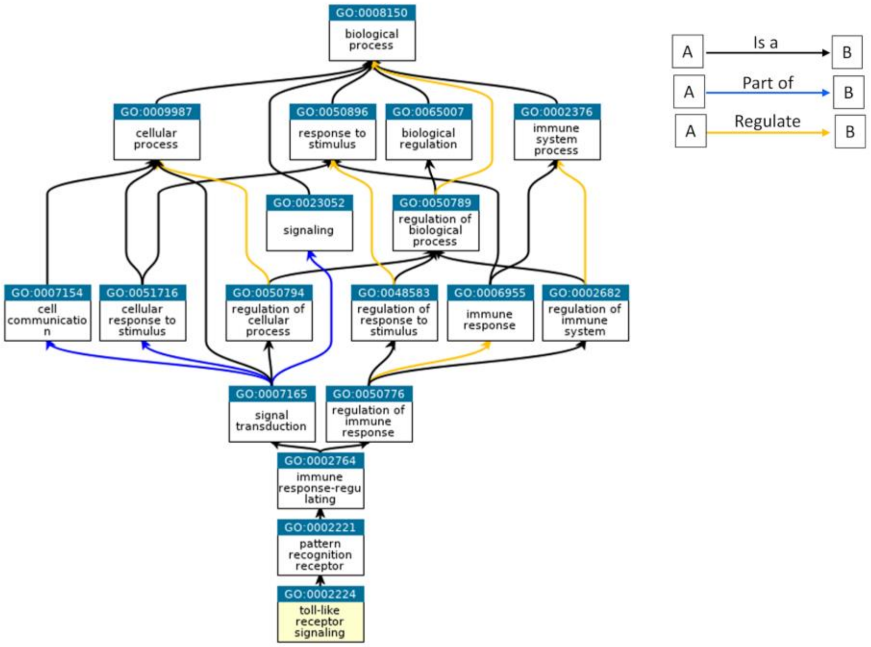

Supplementary Figure S5. Toll-like receptor signaling biological process through the gene ontology clustering by DIANA tool (TarBase algorithm) using the list of the six EV-miRs silenced during the therapy in the responding patients.
